# Contrast-Induced Nephropathy After Computed Tomography in Stable CKD Patients With Proper Prophylaxis: 8-Year Experience of Outpatient Prophylaxis Program

**DOI:** 10.1097/MD.0000000000003560

**Published:** 2016-05-06

**Authors:** Sehoon Park, Myoung-Hee Kim, Eunjeong Kang, Seokwoo Park, Hyung Ah. Jo, Hajeong Lee, Sun Moon. Kim, Jung Pyo. Lee, Kook-Hwan Oh, Kwon Wook. Joo, Yon Su. Kim, Dong Ki. Kim

**Affiliations:** From the Department of Internal Medicine, Seoul National University College of Medicine, Seoul (SP, EK, SP, HAJ, HL, K-HO, KWJ, YSK, DKK); Department of Dental Hygiene, College of Health Science, Eulji University, Gyeonggi-do (M-HK); Department of Internal Medicine, Chungbuk National University Hospital, Chungcheongbuk-do (SMK); Department of Internal Medicine, Seoul National University Boramae Medical Center (JPL); and Kidney Research Institute, Seoul National University, Seoul, Korea (KWJ, YSK, DKK).

## Abstract

Conflicting data have been reported on the clinical significance of contrast-induced nephropathy after CT scan (CT-CIN). In addition, the epidemiologic characteristics and clinical outcomes of CT-CIN following proper prophylactic intervention remain elusive.

We examined the incidence, risk factors, and outcomes of CT-CIN in stable chronic kidney disease (CKD) patients using data collected from our outpatient CT-CIN prophylaxis program conducted between 2007 and 2014. The program recruited patients with an estimated glomerular filtration rate (eGFR) <60 mL/min/1.73 m^2^ using an electronic health record-based pop-up alert system and provided an identical protocol of CIN prophylaxis to all patients.

A total of 1666 subjects were included in this study, and 61 of the 1666 subjects (3.7%) developed CT-CIN. Multivariate analysis showed that baseline eGFR, diabetes mellitus, and low serum albumin were significant risk factors for CT-CIN. The generalized additive model analysis revealed a nonlinear relationship between the baseline eGFR and the risk of CT-CIN. In this analysis, the risk of CT-CIN began to increase below an eGFR threshold of 36.8 mL/min/1.73 m^2^. To assess the outcomes of CT-CIN, patients with and without CT-CIN were compared after propensity score-based 1:2 matching. CT-CIN did not increase the mortality rate of patients. However, patients with CT-CIN were significantly more likely to start dialysis within 6 months of follow-up, but not after those initial 6 months.

CT-CIN developed in only a small number of stable CKD patients who received proper prophylactic intervention, and the risk of CT-CIN was increased in patients with more advanced CKD. Despite the low incidence, CT-CIN conferred a non-negligible risk for the initiation of dialysis in the acute period, even after prophylaxis.

## INTRODUCTION

Contrast-induced nephropathy (CIN) is one of the most common causes of iatrogenic kidney injury in current medical practice.^1^ Although CIN is one of the disease categories of acute kidney injury, unique criteria defined as an increase in serum creatinine (sCr) level of >0.5 mg/dL or >25% from baseline within 48 to 96 hours after use of contrast has been used.^2^ CIN defined by this criteria is associated with adverse outcomes such as hospitalization, mortality, and dialysis.^3,4^ With this in mind, clinical practice guidelines suggest prophylactic interventions using 0.9% saline alone or 0.9% saline plus *N*-acetylcysteine to prevent CIN in patients with renal dysfunction, although there has been debate on the use of *N*-acetylcysteine.^2,5–9^

Because many previous studies examining the risk factors and prognosis of CIN involved contrast agents administered via an arterial route, there has been skepticism about whether those results can be extrapolated to the intravenous contrast agents used in computed tomography (CT). Owing to the lower volume of CT contrast agents and their dilution before reaching the kidneys, some researchers have assumed that CT scans are safer than procedures involving intra-arterial infusion.^10^ Moreover, several recent studies have shown that the incidence of renal dysfunction after contrast-enhanced CT scan was not significantly different from that after unenhanced CT scan.^11–14^ However, doubts remain about the clinical impact of CIN after contrast-enhanced CT (CT-CIN) for several reasons. First, the studies that understated the clinical significance of CT-CIN did not address CIN prophylaxis, which may have been performed in the contrast-enhanced CT group; ^11–14^ thus, the true incidence of CT-CIN after proper prophylaxis needs further study. Second, several previous studies were suspected to overestimate the outcome of CT-CIN because most of the enrolled patients were already hospitalized and had multiple confounding medical conditions.^13–16^ Third, there have been no data reported concerning patient-oriented outcomes following prophylactic management for CT-CIN. Indeed, most of the studies that examined patient-oriented outcomes did not consider whether or how the patients received prophylactic intervention, as stated above.^13,15,16^ In addition, the risk factors for CT-CIN after proper prophylactic intervention remain elusive. In particular, the threshold value for baseline renal function that is associated with an increased incidence of CT-CIN after CIN prophylaxis has not been well established. In this study, we analyzed data from our outpatient CIN prophylaxis program,^4^ which used a standardized protocol of 0.9% saline infusion and *N*-acetylcysteine, to assess the incidence, risk factors, threshold renal function, and patient-oriented outcomes of CT-CIN in stable patients with CKD.

## MATERIALS AND METHODS

### Ethics Statement

This study was approved by institutional review board by Seoul National University Hospital (1503–042–654). This study was conducted in accordance with the principles of the Declaration of Helsinki. As the study was retrospective study without medical intervention, informed consent was waived for electrical health record (EHR) review.

### CIN Prophylaxis Program

Seoul National University Hospital began an outpatient CIN prophylaxis program in January 2007.^4^ Patients with an estimated glomerular filtration rate (eGFR) <60 mL/min/1.73 m^2^ who were scheduled for outpatient contrast-enhanced CT scans were enrolled in the program using an EHR-based alert system and received a standardized CIN prophylactic intervention. In the program, patients visited the daycare center and had a blood sample drawn if no baseline serum creatinine (sCr) level was available within the previous 2 weeks. Patients were referred to the emergency department if any other acute medical condition was identified by the attending physician. For CIN prophylaxis, 1 L of 0.9% saline was administered to the patient; the patient received 0.5 L for 3 hours before the CT scan and an additional 0.5 L for 3 hours after the examination. Additionally, oral *N*-acetylcysteine 300 mg was given 2 times a day for 2 days, starting on the day of CT. A follow-up blood sample that included sCr analysis was performed 48 to 96 hours after the CT scan.

### Study Subjects

The inclusion criteria were age 18 years or above, performance of an outpatient contrast-enhanced CT scans with prophylactic intervention, and an eGFR <60 mL/min/1.73 m^2^. The following patients were excluded from the study: patients who had a history of renal replacement therapy (RRT) before the CT scan; patients who did not have a follow-up sCr analysis performed 48 to 96 hours after the CT scan; patients who were exposed to another contrast agent within 1 month; patients who were admitted to an emergency room or other ward because of a cause other than CIN before follow-up measurement of sCr. In cases with repeated CT scans in a single patient, we included only the most recent study.^13,14^

### Data Collection

We collected the following demographic, laboratory, and clinical information from the EHR review: age, sex, weight, height, body mass index (BMI), baseline and follow-up sCr and eGFR values calculated using the Modification of Diet in Renal Disease (MDRD) equation,^17^ hemoglobin level, serum albumin level, contrast agent type, volume and iodine dosage used, histories of diabetes mellitus, hypertension, congestive heart failure requiring hospitalization, cancer, and liver cirrhosis, and previous use of statins, angiotensin-converting enzyme (ACE) inhibitors, and angiotensin receptor blockers (ARBs).

### Outcome Measurement

The primary outcome was the development of CT-CIN. Laboratory results obtained within 2 weeks before the CT scan were used as the baseline. CIN was defined as an increase in the sCr level of ≥0.5 mg/dL or ≥25% from baseline within 48 to 96 hours after CT.^2,3^ The secondary outcomes were the patient-oriented outcomes of death and the initiation of RRT. The follow-up period was measured beginning on the day of the CT scan and continued until the study ended, the patient died or the patient was lost to follow-up.

In addition to the EHR review, national databases were reviewed to identify outcome events that occurred outside of our hospital. In South Korea, every patient who receives RRT is assigned a diagnosis code for national insurance support. These data are recorded in the RRT registry by the Korean Society of Nephrology (www.ksn.or.kr). Additionally, the death registry maintained by the Statistics Korea (www.kostat.go.kr) tracks the date of death of all Korean people. These sources were reviewed, and relevant data concerning our study patients were included in our analysis after approval by the institutional review board and the Statistics Korea.

### Statistical Analysis

Data are presented as frequencies and percentages for categorical variables and were analyzed using *χ*^2^ tests. Continuous variables were expressed as the mean (standard deviation) or median scores (interquartile ranges) depending on the results of Shapiro-Wilk normality test and were compared using an analysis of variance according to the levels of the baseline eGFR. To identify the risk factors for CT-CIN, logistic regression analysis was performed. We used generalized additive models (GAMs) to explore the relationship between the risk of CIN with a binomial distribution and the baseline eGFR with a smoothing function.^18^ To estimate the eGFR threshold, we first selected the eGFR range and then fitted the GAM to quantify the relationship between eGFR and the risk of CIN. We used Akaike's information criterion (AIC) as a primary measure of model fit. For the AIC, lower numbers within the data set indicate a better model fit. Secondary outcomes were compared between the CIN and the no CIN group. In addition, secondary outcomes in the early period were analyzed by assessing the outcomes during the initial 6 months of follow-up after the CT scan. The long-term effect of CIN was assessed by analyzing secondary outcomes in the late period by excluding cases with a follow-up duration of <6 months. For the subgroup analysis, a baseline eGFR of 30 mL/min/1.73 m^2^ was used as the criterion for dividing the subgroups.

Propensity score matching was performed to compare patients of similar demographic and clinical characteristics for CT-CIN using the MatchIt package in R. Subjects were matched using 1:2 nearest neighbor matching without replacement. Subjects with missing baseline characteristics were excluded from the matching. The following variables were used in propensity score matching: age, sex, total contrast volume used during CT, serum albumin level, baseline eGFR, history of hypertension, diabetes mellitus, cancer, or liver cirrhosis, and the use of statins, ACE inhibitors, or ARBs. Matched results were then compared using the Wilcoxon rank sum test or Student *t* test for continuous variables according to the normality of the data or the *χ*^2^ test for categorical variables.

Overall survival was assessed using a Kaplan-Meier survival curve. The Cox regression proportional hazard method was used to evaluate the secondary outcomes after adjustment for age, sex, contrast volume, and variables that were significant in the multivariate logistic regression risk factor analysis. All statistical analyses were performed using SAS version 9.4 (SAS Institute, Cary, NC) or R package version 3.2.2 (R Development Core Team, Austria). Two-sided *P* values with a statistical significance level of 0.05 were used.

## RESULTS

### Study Population

**FIGURE 1:**
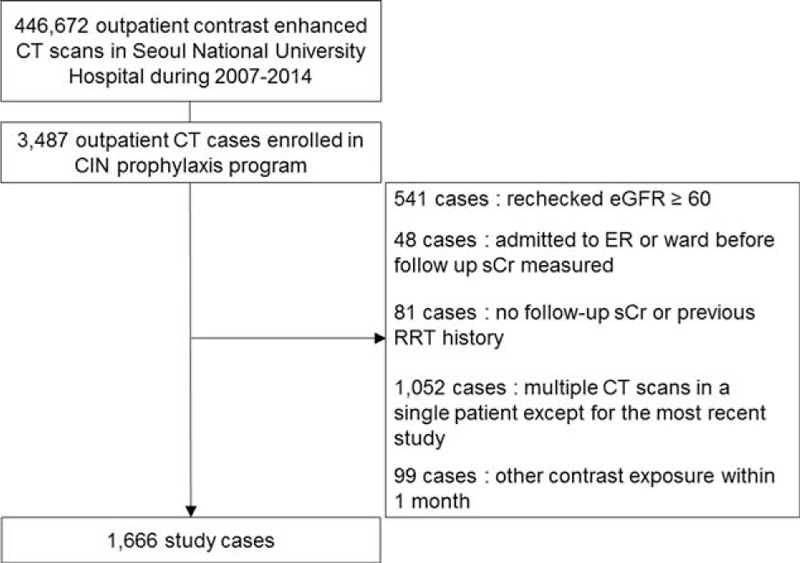
Flow diagram of the study populations.

A total of 446,672 contrast-enhanced CT scans were performed in the outpatient setting from January 2007 to December 2014. Among those CT scans, 3487 CT examinations were performed on patients who received the CIN prophylaxis protocol. After the exclusion criteria were applied, 1666 patients with same number of contrast-enhanced CT scan were enrolled in this study (Figure 1). Mean follow-up duration of patients was 26.5 months.

### Baseline Characteristics and Incidence of CT-CIN

Baseline clinical and laboratory characteristics, stratified by baseline eGFR, are listed in Table 1. Age, sex, history of diabetes mellitus or hypertension, use of ACE inhibitors or ARBs, hemoglobin level, and albumin level showed significant differences across the eGFR groups. CT-CIN developed in 61 cases (3.7%). The rates of CT-CIN were 2.4% (20/837), 2.4% (14/579), and 10.8% (27/250) in the groups with eGFRs of 60 to 45, 45 to 30, and <30 mL/min/1.73 m^2^, respectively.

**TABLE 1:**
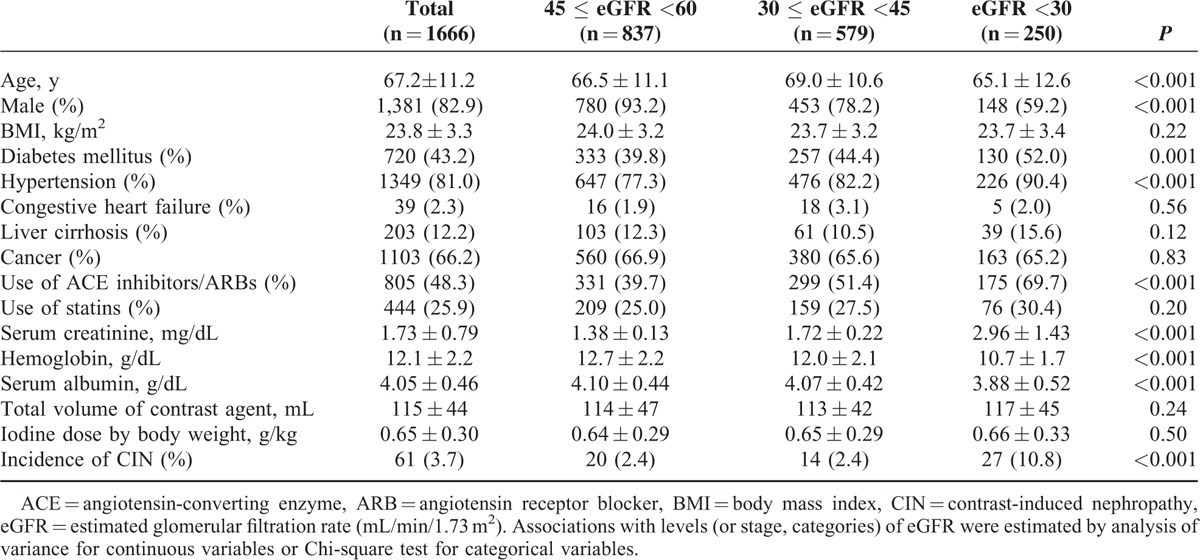
Baseline Characteristics and Incidence of Outcomes According to Baseline Estimated Glomerular Filtration Rate

### Risk Factors for CT-CIN

Univariate logistic regression analysis revealed that sex, diabetes mellitus, liver cirrhosis, baseline kidney function, total volume of radiocontrast agents, and serum albumin level were significantly different between the patients with and without CT-CIN. Multivariate logistic regression analysis was performed using the following variables as covariates (Table 2): age, sex, baseline eGFR, serum albumin level, history of diabetes mellitus, hypertension, congestive heart failure requiring admission, liver cirrhosis, or cancer, total volume of radiocontrast agent, and the use of ACE inhibitors, ARBs or statins. The multivariate analysis revealed that baseline eGFR (odds ratio [OR], 0.955; *P* < 0.001), a history of diabetes mellitus (OR, 2.583; *P* = 0.008), and the serum albumin level (OR, 0.449; *P* = 0.005) were associated with an increased risk of CT-CIN.

**TABLE 2:**
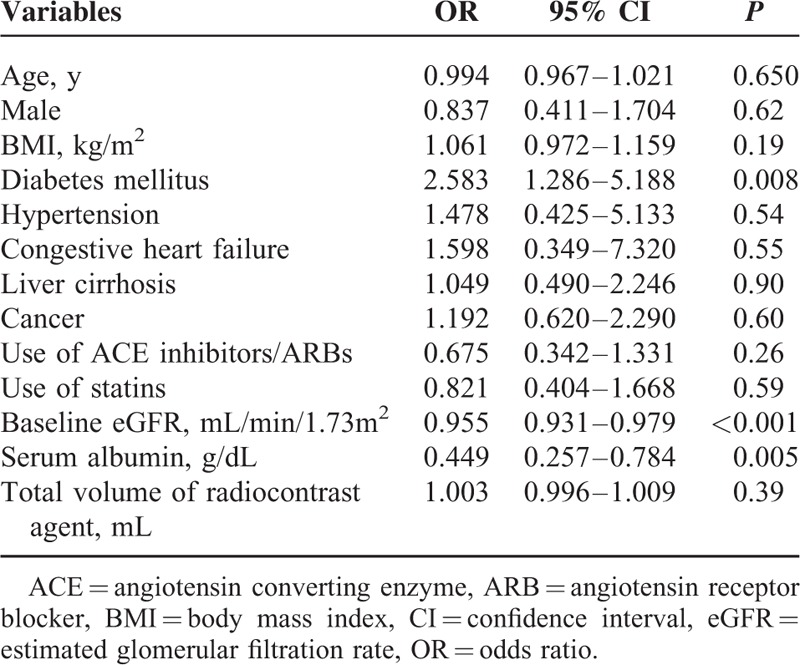
Multivariate Analysis of Risk Factors for Contrast-Induced Nephropathy

### Threshold eGFR Value for CT-CIN

**FIGURE 2:**
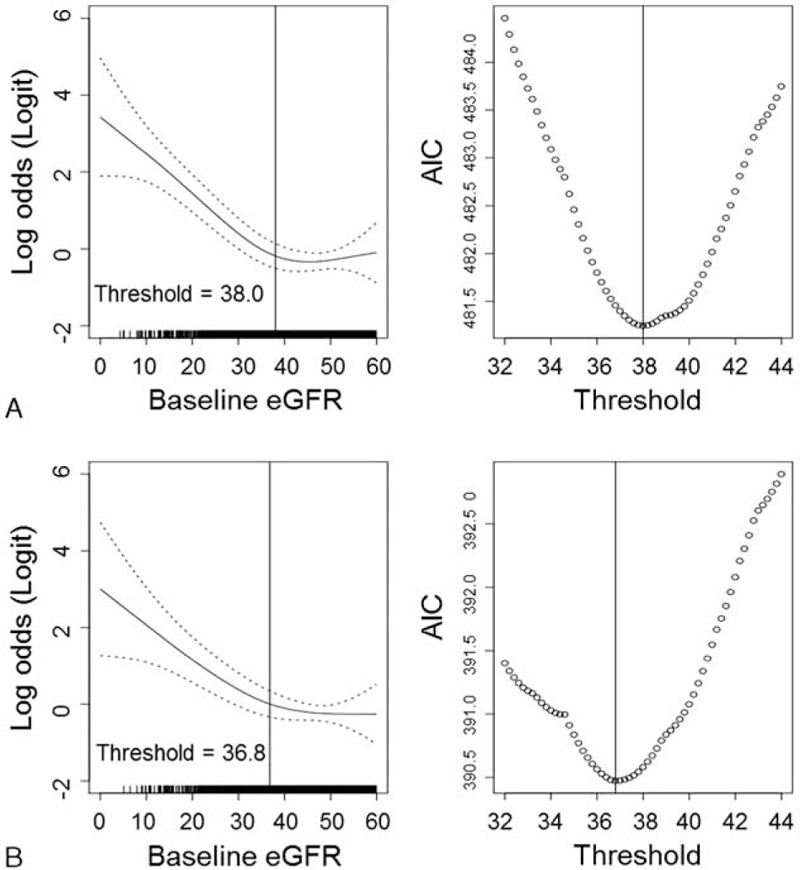
GAMs using binomial distribution for analysis of the threshold baseline kidney function for increased risk of CT-CIN. In the univariate model (A), the smoothing function showing the association between eGFR and the log odds of CIN risk is displayed. In the multivariate GAM analysis (B), the association was adjusted for the following variables: age, sex, BMI, history of diabetes mellitus, history of hypertension, use of statins, ACE inhibitors or ARBs, and serum albumin level. The dotted line indicates the 95% confidence intervals of the logistic odds ratio of CIN risk. The right graphs show the calculated AIC of each GAM plot according to the baseline eGFR. ACE = angiotensin converting enzyme, ARB = angiotensin receptor blocker, BMI = body mass index, CI = confidence interval, CT-CIN = contrast-induced nephropathy, eGFR = estimated glomerular filtration rate, GAMS = generalized additive models.

Because the baseline eGFR was a significant risk factor for CT-CIN and the incidence of CT-CIN markedly increased in patients with a baseline eGFR <30 mL/min/1.73 m^2^, we used a GAM to assess whether a nonlinear relationship existed between eGFR and the risk of CT-CIN and to explore the threshold baseline eGFR that conferred an increased risk of CT-CIN. The result of the GAM and calculated AIC values according to eGFR are shown in Figure 2. Baseline eGFR and the risk of CT-CIN showed a nonlinear relationship after adjustment for age, sex, BMI, history of diabetes mellitus, history of hypertension, serum albumin level, and the use of statins, ACE inhibitors, or ARBs. AIC was used as the primary measure of model fitness. The multivariate-adjusted GAM estimated the eGFR threshold to be 36.8 mL/min/1.73 m^2^, suggesting that the risk of CT-CIN began to increase as the baseline eGFR decreased below that threshold.

### Outcomes of CT-CIN

**FIGURE 3:**
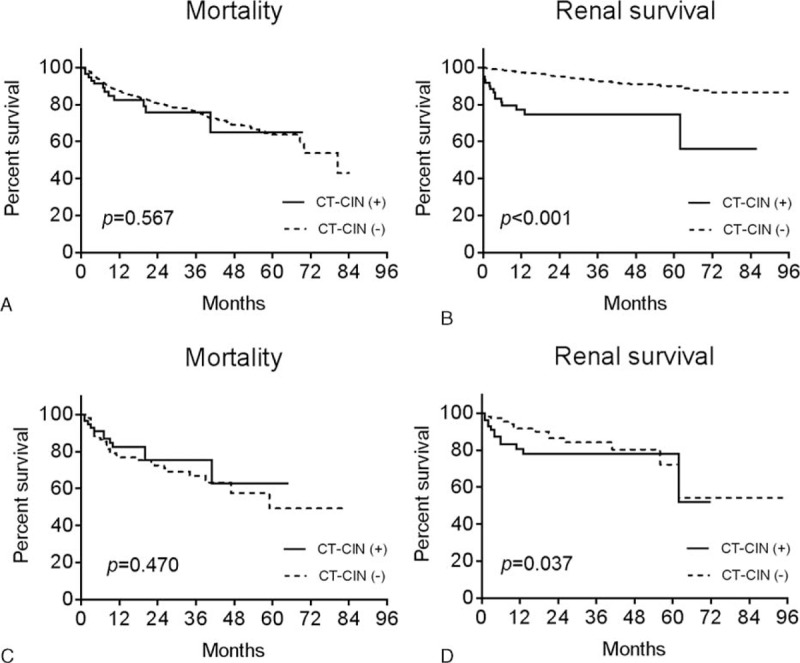
Kaplan-Meier survival curves of mortality (A, C) and renal survival (B, D) before (A, B) and after (C, D) propensity score matching. The X-axis shows the duration from the CT scan by month, and the Y-axis shows the percent survival.

To evaluate the clinical significance of CT-CIN, we analyzed mortality and renal survival before and after propensity score matching. The propensity scores of the baseline characteristics were calculated, followed by 1:2 matching. The matched dataset included 59 patients who developed CT-CIN and 118 patients who did not develop CT-CIN (Table 3). No significant differences in risk factors between the groups were found in the matched dataset. Among them, 44 cases of deaths and 31 cases of start of RRTs occurred. Before and after matching, the overall mortality and renal survival were assessed using a Kaplan-Meier survival curve (Figure 3). In addition, adjusted Cox regression hazard models were constructed to analyze the effect of CT-CIN on patient outcomes (Table 4). First, when we analyzed the entire cohort, CT-CIN did not affect patient mortality (hazard ratio [HR], 1.05; 95% confidence interval [CI] 0.58–1.91; *P* = 0.86), but it significantly affected the initiation of RRT (HR 2.75; 95% CI 1.52–4.98; *P* = 0.001). After discovering the effect of CT-CIN on renal survival, we then assessed renal survival according to the follow-up period. We found that CT-CIN was a significant risk factor for the initiation of RRT within 6 months of the CT scan (HR 4.54, 95% CI 1.93–10.71; *P* = 0.001), but it did not affect the initiation of RRT after 6 months (HR 1.73; 95% CI 0.62–4.81; *P* = 0.30). In the subgroup analysis according to baseline eGFR, CT-CIN was a risk factor for RRT in both groups, which was determined using an eGFR threshold of 30 mL/min/1.73 m^2^. This result was nearly the same in the 1:2 propensity score-matched dataset. In our matched cohort, CT-CIN was not related to patient mortality (HR 0.90; 95% CI 0.46–1.76; *P* = 0.75), regardless of the follow-up period or baseline eGFR. CT-CIN did affect the initiation of RRT (HR 3.05; 95% CI 1.43–6.47; *p* = 0.003), and this trend was observed in both eGFR subgroups, although it was not statistically significant in the group with an eGFR ≥30 mL/min/1.73 m^2^. Similar to the results observed for the entire cohort, the effect on the initiation of RRT differed with regard to follow-up period. CT-CIN affected the initiation of RRT within the first 6 months of follow-up (HR 8.61; 95% CI 2.28–32.61; *P* = 0.002). However, a statistically significant relationship was not observed with the initiation of RRT after the initial 6 months after CT (HR 1.15; 95% CI 0.0.34–3.86; *P* = 0.83).

**TABLE 3:**
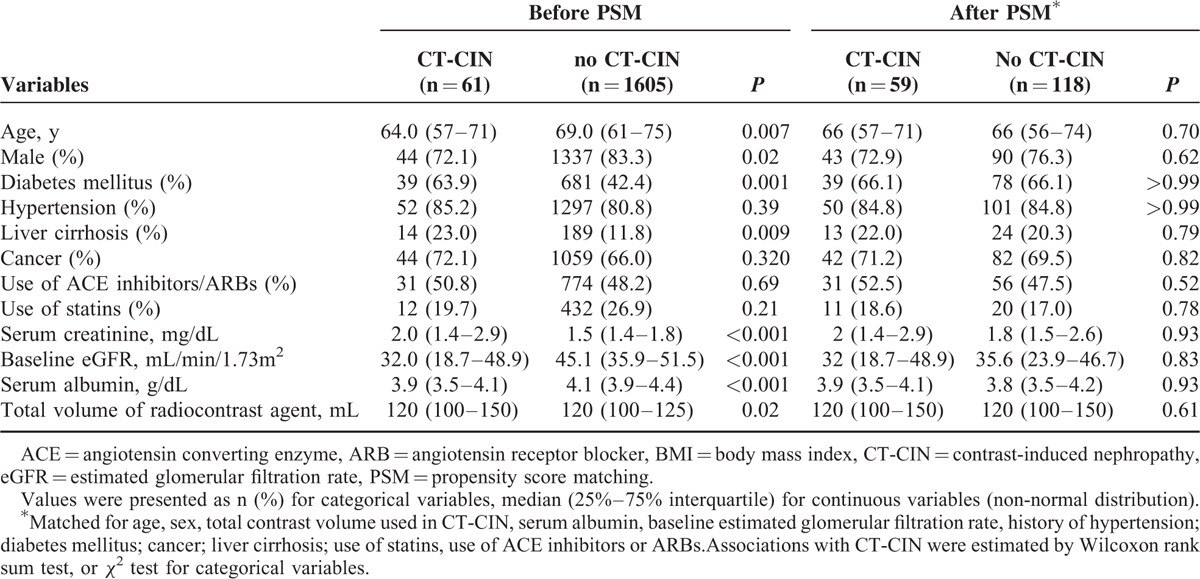
Baseline Characteristics According to Event of Contrast-Induced Nephropathy After CT Scan Before and After Propensity Score Matching

**TABLE 4:**
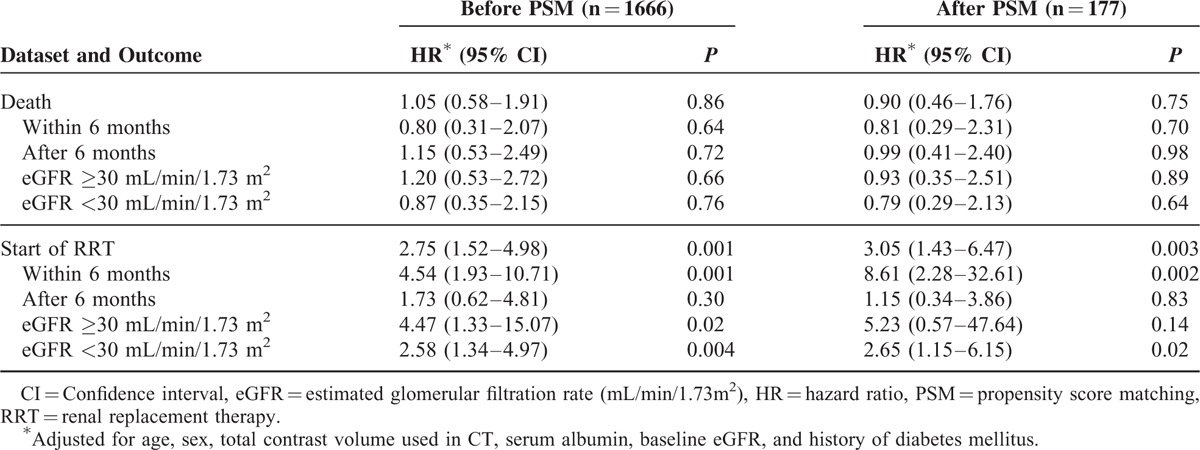
Adjusted HRs (95% CIs) of Contrast-Induced Nephropathy After CT Scan Predicting Mortality and Renal Survival Before and after Propensity Score Matching

## DISCUSSION

One strength of our study was that we analyzed outcomes of CT-CIN following a standardized prophylactic intervention. In addition, most of the CT examinations in our study were performed on patients in stable medical condition because patients with other acute medical conditions were excluded from the study. Consequently, the incidence of CT-CIN in our cohort was much lower than in previous studies, which primarily examined outcomes of hospitalized patients.^13,15,16^ Moreover, mortality and RRT were assessed before and after propensity score matching to minimize any bias from confounding medical conditions.

Several recent studies have asserted that the effects of intravenously administered contrast agents used in CT scans are negligible and that the clinical impact of CT-CIN is insignificant.^11–14^ However, careful interpretation of the results of these studies is necessary, and important limitations should be noted.^19^ Because most clinicians would not prescribe contrast agents to unstable patients, the patients who underwent non-enhanced CT scans may show exaggerated adverse outcomes. This selection bias could lead to the conclusion that acute kidney injury after non-contrast CT scans show comparable patient-oriented outcome with contrast-enhanced CT scans. In addition, as mentioned above, previous studies lacked information concerning CT-CIN prophylaxis measures, which could significantly affect the follow-up levels of sCr. Therefore, the patients who underwent contrast-enhanced CT scans in these previous studies could not have represented CKD patients with CIN prophylaxis intervention.

Previous studies have shown that patients with an eGFR <45 mL/min/1.73 m^2^ have a markedly increased incidence of CT-CIN.^4^ In our study, we demonstrated a nonlinear relationship between the baseline eGFR and the risk of CT-CIN. The threshold baseline renal function associated with an increased risk of CT-CIN was an eGFR of 36.8 mL/min/1.73 m^2^, which suggests that CT-CIN might occur predominantly among patients with more advanced kidney dysfunction. In recent clinical guidelines, an eGFR of 45 mL/min/1.73 m^2^, rather than the previously used eGFR of 60 mL/min/1.73 m^2^, has been suggested as the probable threshold value for CT-CIN risk; our results support this opinion.^2,6^

The studies indicating that CT-CIN was related to increased mortality were questioned for possible confounding bias made by the patients’ coexisting medical conditions.^6,19,20^ Here, we tried to demonstrate the sole effect of CT-CIN by conducting our study in an outpatient setting and by using a matched analysis. Our results showed that CT-CIN was not related to patient mortality in stable CKD patients after proper prophylaxis, regardless of the baseline kidney function and the time since the CT scans. In contrast, CT-CIN was related to an increased risk of RRT initiation in the acute period, even after evidence-based prophylaxis protocol and close monitoring. Because our study participants had preexisting chronic kidney disease, this finding would be accepted and has been demonstrated in many previous studies. However, the long-term outcome of CIN has rarely been described. We showed that CT-CIN was not related with RRT initiation after 6 months, implying that the effect of the contrast agent is limited to the early post-procedure period with patients who recovered from the initial damage.

Our study has several limitations because of its observational nature. First, we could not have included patients who underwent non-contrast CT scans as a control group owing to small number of patients who had their follow-up blood samples few days after non-contrast CT scans. This small number of eligible patients was probably because of their stable medical condition. Also, when considering a study with prospective design, it is nearly impossible to randomize patients or avoid selection bias because of clinical necessity and the potential harm caused by contrast agents. Additionally, the effect of the prophylaxis might contaminate the result. Second, every patient received prophylactic intervention to prevent CT-CIN; thus, the result of our study might not be applicable to patients who undergo contrast-enhanced CT scans without prophylaxis. Moreover, careful interpretation of the results is necessary when using different prophylaxis protocol. Third, we did not study the effect of CT-CIN on medically unstable or hospitalized patients, so the results of our study might not be identically applied to those patients. However, as previously mentioned, reducing the confounding bias would be challenging. To overcome this challenge, further study is necessary and should include hospitalized patients with exclusion of other causes for firm diagnosis of CIN, rather than defining CT-CIN merely by elevation of serum creatinine after contrast-enhanced CT scans.

In conclusion, the incidence of CT-CIN was low among medically stable CKD patients who received proper prophylaxis; the risk of CT-CIN began to increase among patients with late stage 3 CKD. Despite the low incidence, CT-CIN was associated with renal failure requiring RRT in the acute post-procedure period. However, this adverse effect was not observed after 6 months. Mortality was not affected by CT-CIN following proper prophylaxis in stable CKD patients visiting outpatient clinics. Overall, careful prophylaxis and early follow-up visit should be scheduled for CKD patients who undergo CT scans. Also, frequent monitoring of possible renal function deterioration during the acute post-procedure period should be encouraged.
